# Optical and Electrochemical Biosensors Using Electrochemically Etched Porous Silicon

**DOI:** 10.3390/bios16090498

**Published:** 2026-09-06

**Authors:** Teodora Despotovski Kiš, Marko Radović, Brankica Kartalović, Nikola Knežević

**Affiliations:** BioSense Institute, University of Novi Sad, Dr Zorana Djindjica 1, 21000 Novi Sad, Serbia; teodora.despotovski@biosense.rs (T.D.K.); marrad@biosense.rs (M.R.); brankica.kartalovic@biosense.rs (B.K.)

**Keywords:** porous silicon, biosensors, optical sensors, electrochemical sensors, microfluidics

## Abstract

Versatile nanostructured materials based on electrochemically etched porous silicon (pSi) are being developed, which have tuneable pore morphology and unique optical and electrochemical properties that enable their effective biosensing applications. It has been shown that fabrication parameters critically influence pore formation and sensor performance, yet challenges remain in reproducible synthesis, structural stability and device integration. Here we review the electrochemical etching synthesis of pSi and recent advances in pSi-based optical and electrochemical biosensors for detecting bacteria, biomolecules, and viruses. We highlight strategies such as surface functionalisation, incorporation of nanomaterials, and integration with microfluidic and lab-on-a-chip technologies that enhance sensitivity and response times by addressing mass transfer limitations. These developments highlight pSi’s potential as a low-cost, adaptable biosensing material with applications in clinical diagnostics and environmental monitoring, while mapping future directions to overcome current fabrication and stability challenges.

## 1. Introduction

Biosensors play a crucial role in clinical diagnostics, environmental monitoring, food safety, and biomedical research by enabling the rapid, selective, and sensitive detection of biological and chemical analytes. The growing need for portable, low-cost, and highly sensitive sensing platforms has spurred extensive research into nanostructured materials with tuneable physicochemical properties [1,2]. Among these materials, porous silicon (pSi) is particularly notable for its large surface area, controllable pore morphology, biocompatibility, and unique optical and electrical characteristics [3].

Porous silicon is typically produced by electrochemical etching of crystalline silicon wafers in hydrofluoric acid-based electrolytes [4]. This fabrication process enables precise control over pore size, porosity, layer thickness, and surface chemistry by adjusting parameters such as current density, electrolyte composition, etching duration, illumination, and wafer doping. Consequently, pSi structures can be engineered to exhibit microporous, mesoporous, or macroporous morphologies, each with distinct optical and electrical properties suitable for various biosensing strategies [5].

The high surface-to-volume ratio of pSi, along with the presence of two main types of surface moieties (Si-H and Si-OH, as spontaneous oxidation of the surface occurs in air after etching), provides abundant active sites for surface modification [6,7]. This enables the immobilisation of biomolecules such as antibodies, enzymes, nucleic acids, and aptamers, which can be used for the selective detection of diverse targets [8,9]. Its tuneable refractive index and photoluminescent properties make pSi especially attractive for optical biosensors employing interferometric, reflectometric, or fluorescence-based transduction. Additionally, the semiconducting nature and electrochemically active surface of pSi enhance its performance in electrochemical sensing platforms by improving signal amplification and charge-transfer efficiency. Combined with the compatibility of silicon-based materials with established microfabrication techniques, these attributes enable the integration of pSi into miniaturised and multiplexed sensing systems.

In recent years, significant advances in pSi biosensor performance have been achieved through the development of novel surface functionalisation methods, nanocomposite structures, and advanced signal processing approaches [10]. Surface modification with metals, polymers, graphene derivatives, and biomolecular recognition layers has significantly improved sensor stability, selectivity, and sensitivity. Furthermore, the integration of pSi into microfluidic and lab-on-a-chip devices has addressed limitations related to analyte transport and diffusion within porous networks, thereby reducing response times and lowering detection limits [11]. These developments have broadened the applicability of pSi-based sensors for detecting various analytes in complex real-world environments.

Despite extensive research on pSi, key challenges remain, including achieving reproducible fabrication, ensuring long-term stability, controlling surface oxidation, and enabling scalable integration into practical sensing platforms. Addressing these challenges requires a comprehensive understanding of how electrochemical etching conditions influence pore formation and the resulting sensing behaviour, which is essential for advancing pSi technologies toward commercial and clinical applications.

Several reviews of pSi-based biosensing are already available in the literature [3,10,11], including surveys of pSi photonic biosensors and of hybrid plasmonic/quantum-dot pSi platforms [12,13,14]. The present review differs from these earlier works in the following aspects: (1) it deliberately covers both optical and electrochemical pSi side by side, within a single fabrication framework (electrochemically etched pSi), so that their relative strengths, sensing mechanisms and mass-transfer limitations can be compared directly; (2) it restricts its literature coverage to the past five years to reflect the current state of the art; and (3) it emphasises mass-transfer and microfluidic integration strategies toward enhancing practical device performance, which is treated only briefly, if at all, in prior reviews of the field.

In this review, we examine the principles and mechanisms underlying the electrochemical synthesis of pSi and how structural parameters influence its properties relevant to biosensing applications. We also summarise recent advances from the past five years in pSi-based optical and electrochemical biosensors for detecting bacteria, biomolecules, and viruses, with a focus on strategies to enhance sensitivity and device performance. Finally, we discuss the integration of pSi with microfluidic and lab-on-a-chip systems, as well as current challenges and future perspectives for the development of next-generation pSi biosensors.

### 1.1. Development of pSi Films

Electronic-grade silicon wafers, with a 99.9999999% (9 N) purity, are typically produced using the Czochralski growth process [15]. In this method, a seed crystal is dipped into a bath of molten polysilicon and slowly withdrawn to form a cylindrical ingot of high-purity monocrystalline silicon. During this process, carefully controlled amounts of donor impurity atoms, such as phosphorus (for n-type) or boron (for p-type), can be introduced to achieve the desired doping and electrical characteristics. These wafers are commonly manufactured with crystal orientations such as (100), (111), and (110).

Fabrication of pSi from Si wafers using electrochemical etching was first reported by Uhlir nearly seven decades ago [16]. Different pSi morphologies and porosities can be achieved by varying formation parameters such as etching current density, electrolyte concentration, bath temperature, and irradiation wavelength/intensity, which in turn affect its properties [17,18,19]. Etching is often performed in organic solvents such as ethanol, acetonitrile, or dimethylformamide containing hydrofluoric acid (HF) to produce structured porosity in silicon wafers [20]. Alternatively, methods such as metal-assisted etching and chemical etching using mixtures of HF and nitric acid have also been widely employed to fabricate diverse pSi nanostructures [21], while safer alternatives, which etch in ionic liquids without HF, are also emerging [22].

The tendency for pores to align with the crystal’s (100) direction is the most noticeable morphological feature in most pSi samples. This is mostly a chemical effect; the durability of the different crystal faces against chemical attack results in what are known as “crystallographic pores.” For instance, hydrogen atoms are connected directly above silicon atoms on silicon’s hydrogen-terminated (111) face, which is therefore the most stable Si-H bond and least susceptible to reaction with HF.

The process of obtaining pSi through etching varies between n- and p-type Si wafers. The main differences are influenced by the doping type, doping level, and specific etching conditions applied to each type of Si wafer. Typical methodologies for the development of biosensors include light irradiation during etching of n-type Si, while electrochemical etching of p-type Si is conducted without exposure to light. This process can produce various morphologies, such as sponge-like structures, depending on the wafer’s resistivity and the specific etching conditions [23].

### 1.2. Mechanism of Pore Formation

**Figure 1:**
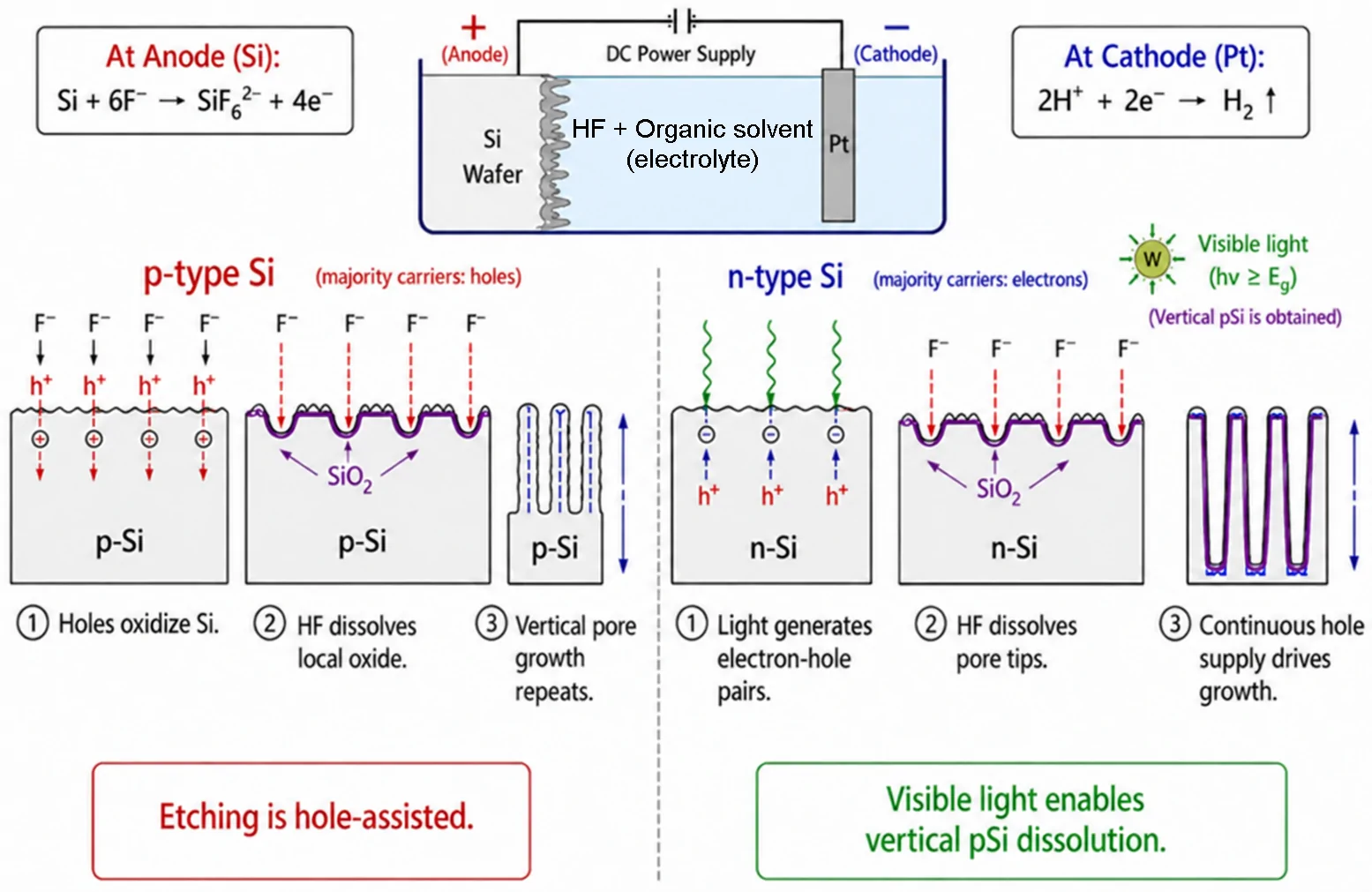
Schematic representation of the electrochemical formation of porous silicon in p-type and illuminated n-type Si.

The pore formation mechanism has been the subject of previous works in detail [21,24,25]. Therefore, we are covering in this section only basic concepts of pore formation. During electrochemical etching, the silicon wafer serves as the anode, where the following etching reaction takes place: Si + 4F^−^ ⟶ SiF_4_ + 4e^−^. When a potential is applied between the cathode (typically Pt) and the silicon anode, a positive potential develops on the Si surface (Figure 1). Variations in the distribution of this potential lead to preferential accumulation of F^−^ ions at certain sites, which in turn determines where pores form. The initially formed SiF_4_ can further dissolve via the reaction: SiF_4_ + 2F^−^ ⟶ SiF_6_^2−^. Electrochemical dissolution of Si in HF requires holes (h^+^) at the Si-electrolyte interface, so the availability and distribution of holes determine pore initiation and growth. Thus, the pore formation mechanism is quite different in n- vs. p-type silicon because of how the necessary holes are supplied for the anodic dissolution reaction. In the case of p-type Si, h^+^ are the major carriers and readily available at the Si surface at anodic bias. As a result, the initiation of pore formation is easy, even in the dark. Pore growth is governed mainly by electric field focusing and HF transport, not hole supply. In the case of n-type Si, hole supply at the Si surface is insufficient under normal anodic bias, and the initiation of pore formation requires illumination, very high anodic bias (strong inversion layer) or high doping to provide enough holes. As illumination leads to pores forming only in the illuminated region, it can be used for micropatterning. On the other hand, high bias can inject holes by band bending but risks electropolishing. Thus, the pores in n-type Si are usually larger (macropores) because the hole distribution is more spread out and less localised than in p-type.

The pore nucleation sites are strongly correlated with the inhomogeneous local distribution of the dopant [21], as well as possible effects of other microscopic inhomogeneities such as surface defects (scratches, dislocations). Namely, dopant distribution in wafers is rarely perfectly uniform due to vertical gradients (from diffusion profiles or epitaxial layers) and lateral fluctuations (from implantation or crystal growth). These variations affect local hole availability during etching. Once a small pit forms, field focusing ensures the etching continues there, evolving into a pore. For n-type silicon, the anodisation bath is typically irradiated, which increases the lifetime of the minority carriers (holes), to promote pore formation and ensure homogeneous pore distribution. In this case, the pores preferentially start at slightly lower dopant concentration zones (or regions where recombination is less intense) [21]. Once a pore starts, field focusing again localises further etching. The progress of electrochemical etching is determined by the relative rates of silicon oxidation and fluoride-assisted dissolution, which is influenced by current density, HF concentration, wafer properties and mass transport [24,25].

## 2. Application of pSi as Optical Biosensors

Optical biosensors can be categorised in numerous ways depending on the underlying physical principles and technological platforms they employ [26]. Within the framework of this review article, pSi-based optical biosensing is classified into three broad categories, mirroring the historical evolution of optical sensing from classical to quantum regimes:

1. Classical optical technologies (reflections, transmission, scattering, RIFTS, SPR, etc).
2. Photonic technologies (pSi photonic crystals, waveguides, resonators, interferometers, etc).
3. Quantum-enabled optical biosensing (quantum dots in pSi).

### 2.1. Classical Optical Technologies (Reflections, Transmission, Scattering, RIFTS, SPR)

Classical optical technologies represent the foundational sensing modality in the field of optical biosensors, exploiting well-established physical phenomena, including reflection, transmission, scattering, resonant interferometric Fourier transform spectroscopy (RIFTS), and surface plasmon resonance (SPR), to transduce molecular binding events at the sensor surface into quantifiable optical signals [27]. A pioneering pSi biosensor reported by Lin et al. employed direct monitoring of Fabry–Pérot interference fringe shifts arising from refractive index changes within the porous layer [28]. Subsequent work included the introduction of RIFTS, in which the reflectance spectrum is transformed by Fourier transform to extract changes in the effective optical thickness (EOT) [29]. Infiltration of analyte molecules into the pores increases the local refractive index, producing a measurable shift in EOT that serves as the transduction signal. This approach enables real-time, label-free monitoring of binding events with high sensitivity and instrumental simplicity, requiring only a broadband light source and a compact spectrometer.

In the domain of plasmonic sensing, pSi has been combined with metallic nanostructures and photonic surface states to achieve SPR-like signal transduction and enhancement. Scattering-based methods, and in particular surface-enhanced Raman scattering (SERS), constitute a distinct and increasingly prominent branch of classical optical sensing on pSi substrates [30,31,32,33]. In SERS-based pSi biosensors, metallic nanoparticles, most commonly silver or gold, are deposited within or onto the pSi porous network, generating plasmonic hotspots that amplify the intrinsically weak Raman scattering signal of analyte molecules by several orders of magnitude. The highly porous structure of pSi provides an ideal scaffold for nanoparticle immobilisation while simultaneously concentrating analyte molecules in close proximity to the plasmonic hotspots, leading to synergistic enhancement.

Achieving low detection limits remains a key objective in the development of pSi biosensors for clinical diagnostics and environmental monitoring. Sensor performance is often constrained by signal noise, particularly when detecting analytes at low concentrations. To address this limitation, Ward et al. introduced a signal-processing strategy based on Morlet wavelet phase analysis (MWPA), which significantly enhanced the performance of pSi optical biosensors [34]. Their approach reduced the limit of detection (LOD) by approximately one order of magnitude compared with conventional signal-processing methods, including RIFTS and interferogram averaging over wavelength (IAW). Further work demonstrated the use of MWPA to enhance spectral noise suppression and phase sensitivity for detection of miRNA-155 with an ultralow limit of detection (~0.13 pM) and high specificity [35].

Beyond improvements in signal processing, advances in surface functionalisation and biorecognition elements have enabled highly selective detection of clinically relevant biomarkers. Graham et al. developed a label-free pSi aptasensor for the detection of anterior gradient homologue-2 (AGR2) [36], a protein biomarker associated with several cancers, particularly pancreatic cancer. The reflectance sensor combined enhanced mass-transfer strategies with MWPA to suppress system noise. As a result, the aptasensor achieved a detection limit of 15 nM in simulated pancreatic juice, despite the presence of competing proteins.

### 2.2. Photonic Technologies (pSi Photonic Crystals, Waveguides, Resonators, Interferometers)

Photonic technologies extend considerably beyond classical optical sensing, exploiting the precise engineering of light–matter interactions at the nanoscale to achieve detection sensitivities and selectivity that classical reflectance or scattering methods cannot readily attain. In the context of pSi biosensors, photonic technologies encompass a broad range of nanostructured optical architectures, including one-dimensional photonic crystals such as Bragg reflectors, rugate filters, and microcavities, as well as waveguides and photonic crystal resonators, all of which exploit the periodic modulation of refractive index within the pSi film to engineer photonic bandgaps, resonant modes, and evanescent field confinement [37]. The combination of a precisely engineered photonic structure with the high internal surface area of pSi creates a powerful synergy: the porous scaffold concentrates analyte molecules within the most optically sensitive regions of the device, while the photonic architecture converts minute refractive index changes into sharply resolved spectral shifts [12].

One-dimensional (1D) photonic crystals based on pSi are fabricated by alternating electrochemical etching conditions to produce periodic stacks of high- and low-porosity layers, resulting in structures with well-defined photonic bandgaps [38]. Among these, the distributed Bragg reflector (DBR) or Bragg mirror [39], is one of the most fundamental and widely employed architectures. In a DBR, alternating quarter-wavelength layers of a high- and low-refractive index produce a high-reflectivity stop band whose spectral position shifts in response to analyte binding within the pores. On the other hand, a pSi microcavity (pSiMC) represents a more advanced photonic architecture, comprising two Bragg mirror stacks flanking a central defect layer that breaks the periodicity of the structure. Light is strongly localised within the defect layer, producing a sharp resonance dip within the photonic stop band that is highly sensitive to changes in refractive index within that region. This localisation makes microcavities among the most sensitive of all pSi photonic architectures. Finally, Rugate filters are a refinement of the DBR concept in which the refractive index is varied sinusoidally rather than abruptly between layers, suppressing higher-order harmonic peaks in the reflectance spectrum and producing a single, narrow, and highly resolved reflectance peak [40]. The peak shifts upon analyte infiltration into the pores provide an unambiguous, low-background optical readout.

The spectral shifts induced by analyte infiltration into porous silicon (pSi) pores provide a label-free optical readout, resulting from changes in the effective refractive index of the porous layer. The broad range of pSi photonic architectures, including Fabry–Pérot films, Bragg reflectors, microcavities, and rugate filters, highlights a versatile design space at the interface of photonic engineering and nanoporous materials science. Through tuneable optical properties, adaptable surface functionalisation, and compatibility with microelectronic and MEMS technologies, these platforms can enable sensitive and selective biosensing. Their integration with advanced recognition chemistries, miniaturised readout systems, and multiplexed sensing formats further supports their potential for point-of-care, wearable, and implantable diagnostic applications [3,41].

### 2.3. Quantum-Enabled Optical Biosensing (Quantum Dots in pSi)

Quantum-enabled optical biosensing represents the most advanced frontier within the optical pSi biosensing landscape, encompassing detection strategies that explicitly exploit quantum mechanical phenomena—including quantum confinement, photoluminescence, Förster resonance energy transfer (FRET), and quantum optical effects—to achieve sensitivity levels and detection mechanisms that classical and photonic approaches cannot readily provide. In the context of pSi-based platforms, Quantum-enabled optical biosensing is realised primarily through the integration of quantum dots (QDs) within pSi nanostructures, including single-layer interferometers, Bragg reflectors, and microcavities, as well as through the intrinsic photoluminescent properties of silicon-based quantum dots themselves [14]. The defining feature of this category is that the transduction signal originates, at least in part, from quantum optical phenomena rather than from purely classical refractive index changes or geometric optical effects. When embedded within or conjugated to pSi photonic architectures, QDs serve a dual function: they act as high-refractive index labels that amplify the classical EOT or resonance shift signal, and simultaneously as quantum emitters whose photoluminescence intensity or spectral position encodes additional analytical information. This dual transduction capability is a hallmark of quantum-enabled biosensing on pSi platforms, allowing simultaneous multimodal readout from a single sensor element.

We use “quantum-enabled optical biosensing” here in a deliberately narrow, operational sense, and we want to be explicit about the distinction, since the term is sometimes used more loosely in the literature. Within the three-tier classification adopted in this review, a device is placed in the classical or photonic categories whenever its transduction signal can be fully explained by classical electromagnetism, i.e., by a change in the bulk or local refractive index of the porous layer (RIFTS, DBR/microcavity resonance shifts) or by classical plasmonic field enhancement (SPR, SERS). A device is placed in the quantum-enabled optical biosensing category only when at least part of its transduction signal instead originates from a quantum-mechanical process specific to the transducer or label itself: the discrete, size-tuneable emission of a semiconductor quantum dot arising from three-dimensional carrier confinement, or the near-field, distance-dependent (Förster) energy transfer between a donor and acceptor chromophore, which is fundamentally distinct from a propagating-wave interference effect. On this basis, a plain RIFTS or DBR sensor that only tracks a reflectance-peak shift remains “photonic” even if it uses a photonic-crystal architecture, whereas a sensor whose readout is the photoluminescence intensity, lifetime or FRET ratio of an embedded quantum dot or fluorophore is classified as “quantum-enabled optical” because that signal cannot be described purely by the effective-medium refractive index picture used for the other two categories.

The quantum-enabled optical biosensing approaches described above are, at present, both the most sensitive and the most experimentally demanding of the three pSi optical biosensing categories. As quantum emitter technologies mature and pSi photonic engineering advances, the boundaries between photonic and quantum transduction modalities are expected to blur further, opening new avenues for ultrasensitive biosensors.

## 3. Overview of Recent Articles on Optical Biosensors

Characteristics of recently reported optical biosensors based on pSi are summarised in Table 1.

### 3.1. Bacterial Detection

The development of biosensors has gained significant attention for rapid and accurate bacterial detection. This addresses the challenge of slow diagnostic methods that hinder timely therapeutic responses, particularly in the context of antimicrobial resistance. New sensing advances based on pSi typically offer rapid, cost-effective, and adaptable platforms for bacterial detection, which could improve diagnostic practices in clinical and environmental settings.

Vercauteren et al. presented a novel pSi membrane (pSiM) biosensor that enables rapid optical detection of *B. cereus* lysate, achieving detection limits as low as 10^5^ colony-forming units per mL (CFU/mL) within one hour [42]. Selectivity of the detection is ensured by using endolysin PlyB221 for selective lysing of *B. cereus*, which is encoded by the bacteriophage Deep-Blue, targeting this bacterial strain. The detection relies on monitoring the changes in EOT occurring through the infiltration of bacterial lysate inside the membrane. The same research group employed atomic layer deposition (ALD) to coat the pSi membranes with titanium dioxide, enhancing their optical properties and stability in aqueous environments [43]. This sensing platform was also applied for detection of bacteria upon selective lysis of target bacteria, which subsequently alters the optical characteristics of the sensor, achieving an improved sensitivity of 10^3^ CFU/mL.

Waterborne diseases such as cholera and travellers’ diarrhoea pose significant threats to public health, necessitating rapid diagnostic tools for effective outbreak management. Antunez et al. presented a regenerable microfluidic biosensing platform that utilises a hybrid material composed of pSi interferometric transducers and thermoresponsive multivalent glycopolymers, achieving detection of *E. coli* heat-labile enterotoxin (LTB) at concentrations as low as 0.135 μM in a label-free format [44]. The authors employed a temperature-mediated “catch-and-release” mechanism, allowing for selective protein capture through pSi/polymer-attached recognition motifs and sensor regeneration over multiple cycles. Another study on detection of *E. coli* employed an indirect immunoassay coupled with a syringe filtration technique to enhance specificity and minimise interference from other pathogens [45]. This approach allowed for real-time monitoring of *E. coli* concentrations in complex matrices, including ground and irrigation water, as well as milk samples.

**Figure 2:**
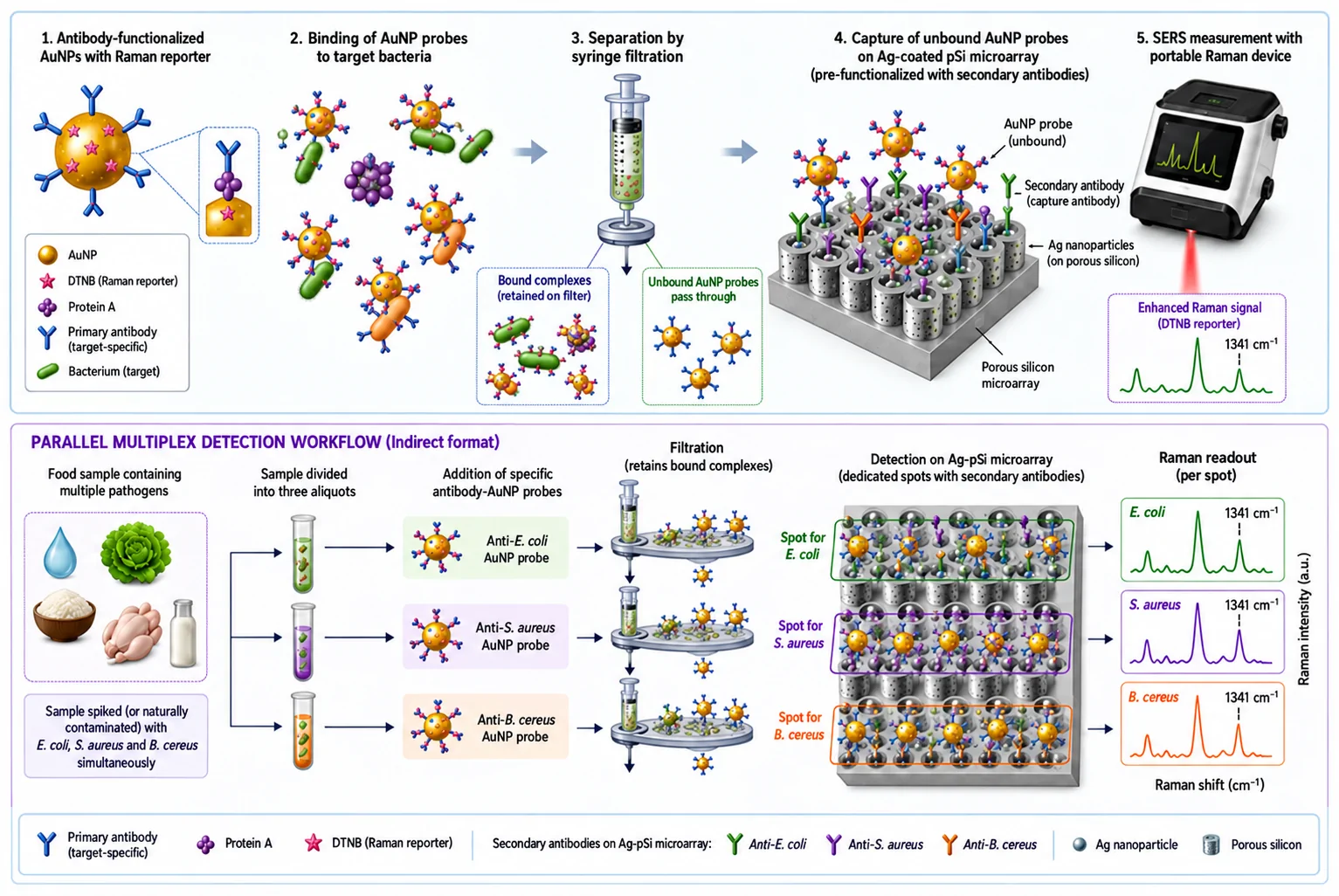
Schematic representation of the SERS-based indirect immunoassay and parallel multiplex detection of *E. coli*, *S. aureus*, and *B. cereus*, based on the sensing strategy reported by Muthukumar et al. [31].

Rapid, on-site detection of foodborne bacteria is essential for food safety, though multiplex detection in complex food matrices remains limited by long assays, procedural complexity and lack of sensitive, field-deployable transducers. Muthukumar et al. present an indirect SERS immunoassay that pairs Raman-tagged (with 5,5′-dithiobis-(2-nitrobenzoic acid), antibody-functionalised gold nanoparticles with a silver-coated pSi (Ag-pSi) microarray to enable rapid, multiplexed detection of *E. coli*, *S. aureus* and *B. cereus* (Figure 2) [31]. Bacteria are captured by distinct AuNP SERS-tags, removed by syringe filtration, and the remaining tags are quantified on an Ag-pSi microarray with a portable 785 nm Raman reader. This indirect microarray amplification strategy reduces reagent use, simplifies multiplexing, and supports pen-side deployment. However, the platform requires three independent antibody-functionalised nanoparticle preparations and three separate sensing channels. The assay is best described as a parallel multiplex microarray rather than a spectrally multiplexed SERS biosensor.

### 3.2. Biomolecule Detection

For detection of small biomolecules, such as glucose, the nanostructured porosity of pSi can be preferred over macroporous pSi samples. This effect was showcased by reflectance measurements on pSi samples with different porosity, while containing physisorbed glucose oxidase (GOX) enzyme for specific binding to the analyte [46]. However, in terms of detecting larger biomolecules, such as heat shock protein 70 (HSP70), pSi with larger pores are preferred due to pore clogging that impedes molecular infiltration [47]. Herein, the authors employed a pSi platform with a pore diameter of 40 nm, covalently functionalised with antibodies, which allowed infiltration of the large protein within the pores and its specific detection through reflectance measurements.

To increase the sensitivity of detection, dual detection methods are being employed, such as utilising a pSi Bragg mirror combined with CdSe/ZnS QD labelling for highly sensitive β-lactoglobulin (β-lg) detection, by measuring both refractive index changes and fluorescence signals [48]. A similar methodology was employed for low-cost detection of DNA [49]. The authors functionalised carboxy-terminated QDs with a single-stranded DNA, while the surface of a pSi Bragg mirror structure was functionalised with a complementary DNA strand. pSi Bragg mirrors were irradiated by a 488 nm laser, and the fluorescence emission was enhanced upon hybridisation and binding of QDs within the functionalised pSi pores. This approach allows for rapid, parallel detection of DNA concentrations, significantly reducing detection time compared to traditional methods. In their following paper, the research group employed the angle spectrum method for reading the change in the redshift of the resonance peak angle before and after the hybridisation of single-stranded DNA-functionalised QDs within the pSi assembly microcavity device, achieving a detection limit of 13.25 pM for target DNA [50]. This approach significantly amplifies the refractive index changes associated with biomolecular interactions, leading to improved detection capabilities.

Another dual-mode sensing platform was successfully developed through the combination of plasmonic nanohole arrays with pSi [51]. Monitoring the optical responses of both channels, namely, SPR (plasmonic nanohole array) and EOT changes (pSi monolayer), was employed upon sequential exposure to solutions of protein A, bovine serum albumin (BSA), functionalised biotin, and rabbit IgG. The sensing response was tested with sucrose as an analyte, demonstrating that plasmonic nanohole arrays on top of pSi monolayers had a sensitivity of 386 ± 5 nm/RIU, while sensors prepared on glass substrates (without SPR channel) had sensitivities of 213 ± 12 nm/RIU.

Kumar et al. first developed a pSi Fabry–Pérot interferometric biosensor for the label-free optical detection of botulinum neurotoxin C through antibody-based recognition [52]. They subsequently advanced this platform into a dual-mode sensing system composed of two adjacent interferometers, enabling simultaneous immunological quantification of the toxin and assessment of its endopeptidase activity [53]. This dual-detection strategy improves analytical reliability while providing information on the biological functionality of the toxin, making the platform more suitable for practical diagnostic and food safety applications.

**Figure 3:**
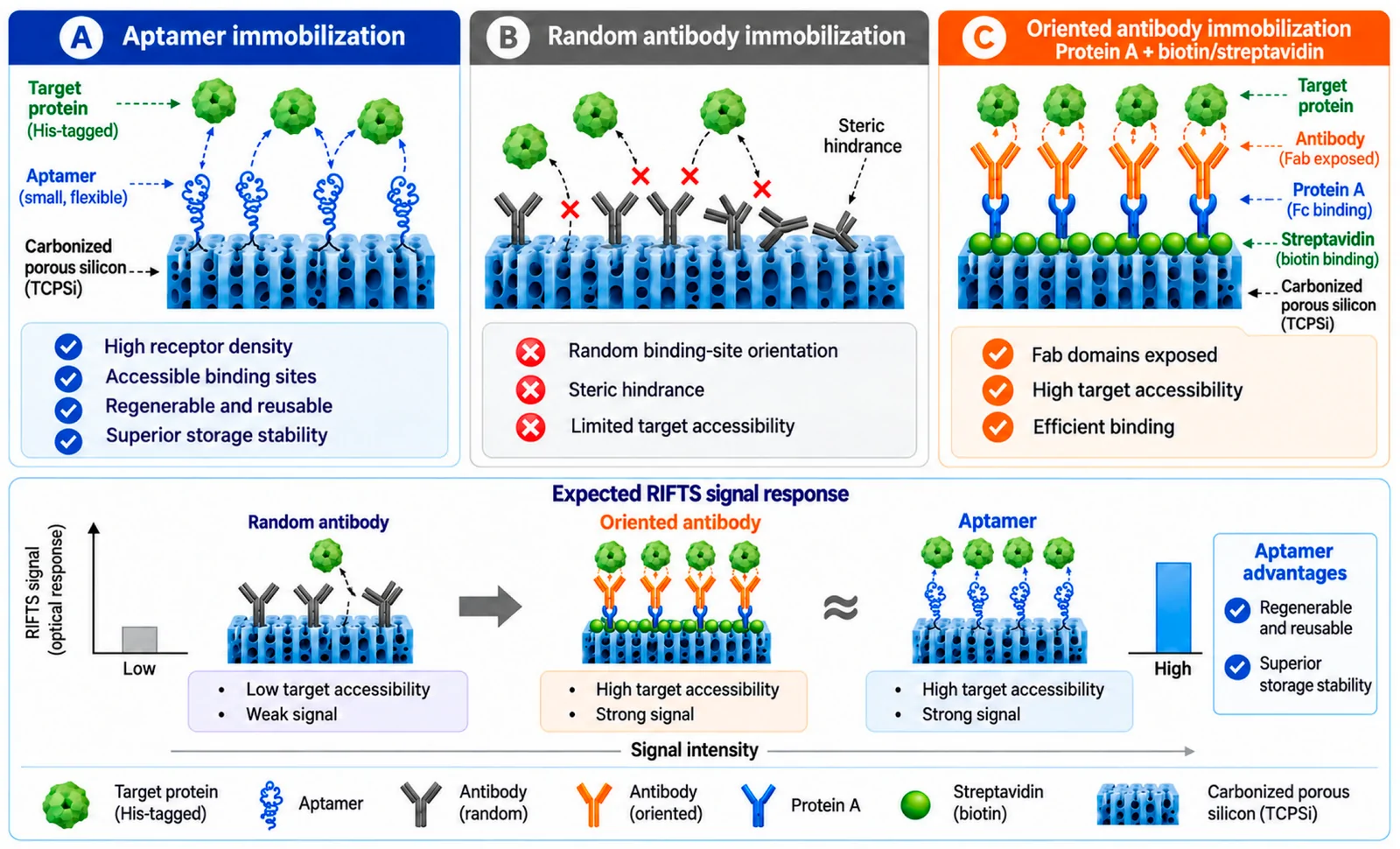
Schematic comparison of aptamer, randomly immobilised antibody, and Fc-oriented antibody functionalisation of porous silicon optical biosensors, illustrating the influence of bioreceptor size and orientation on target accessibility and biosensing performance. The scheme is based on the experimental findings reported by Arshavsky-Graham et al. [54].

Surface functionalisation is another important aspect in the development of effective biosensors. Arshavsky-Graham et al. demonstrated the comparative performance of aptamer-based and antibody-based RIFTS biosensors for a his-tagged protein under equivalent conditions (Figure 3) [54]. The results revealed that the capabilities of antibody-functionalised biosensors severely depend on their orientation on the pSi surface. Furthermore, while aptamer and oriented-antibody biosensors exhibit similar sensitivity and selectivity for detecting a his-tagged protein, the aptamer-based biosensor outperforms in terms of reusability and storage stability.

Di Giulio et al. report an optical sensor based on iminodiacetic acid (IDA) functionalised pSi that chelates and exchanges metal ions (Ni^2+^, Cu^2+^, Zn^2+^, Fe^3+^) to reconfigure target binding [55]. Key findings include effective Cu^2+^-functionalised detection of the dipeptide carnosine (LOD ~25 μM) validated in murine brain extracts, successful switching to Zn^2+^ with comparable performance, and reconfiguration to Fe^3+^ for ATP sensing (LOD ~30 μM), with good linearity, reproducibility, and regeneration.

On the other hand, covalent functionalisation of pSi with a rationally truncated peptide nucleic acid (PNA) probe enables low-cost, high-sensitivity detection of cardiac troponin T (cTnT) [56]. This approach is significant because short PNAs are cost-effective and reduce steric hindrance, while pSi provides a simple, low-cost optical transducer. The 12-mer (PNA-3) retains specific cTnT affinity, achieved stable functionalisation (1.12 ± 0.30 pmol/cm^2^ surface density), and delivered a clinically relevant linear response (0.02–0.16 ng/mL) with LoD ≈ 0.030 ng/mL.

A recent report showcased a novel approach utilising pSi arrays for protein identification and quantification even without the need for capture agents [69]. The authors employed optical reflectance measurements combined with machine learning techniques to classify and quantify three proteins: bovine serum albumin, chicken ovalbumin, and avidin, across a concentration range down to 300 nM. Their method demonstrated 100% accuracy for known concentrations and 87.5% accuracy for unseen concentrations.

### 3.3. Virus Detection

Detection of pathogens like the Chikungunya virus poses significant challenges, particularly regarding the stability of bioreceptors under harsh conditions. Layouni et al. present a novel approach utilising a pSi biosensor for the peptide-based capture of the Chikungunya virus E2 protein [57], demonstrating its effectiveness in both room temperature and elevated temperature scenarios. The authors employed a combination of contact angle measurements, ATR-FTIR spectroscopy, and optical reflectance to confirm successful peptide functionalisation and target protein capture.

Related to cervical cancer diagnostics, the detection of high-risk human papillomavirus (HPV) types 16 and 18 is crucial due to their association with pre-cancerous lesions. Current detection methods face challenges such as low specificity, sensitivity, and lengthy result turnaround times, highlighting a significant knowledge gap in rapid and accurate HPV screening. Rodríguez-Montelongo et al. present a novel pSi biosensor that effectively detects HPV16 and HPV18 through a simple optical method [58]. The authors employed a biocompatible pSi microcavity, functionalised with single-stranded DNA probes, to achieve selective DNA hybridisation, confirmed by reflectance spectrum shifts and fluorescence microscopy. Their findings demonstrate that the pSi biosensor can accurately differentiate between complementary and non-complementary DNA.

### 3.4. Integration of pSi Biosensors

Even though pSi shows promising characteristics for biosensing applications due to its large surface area and unique optical properties, it faces significant mass transfer limitations, which hinder sensitivity and performance compared to planar biosensors. In their study, Arshavsky-Graham et al. present a theoretical model that elucidates the complex mass transport phenomena affecting pSi biosensors, revealing that hindered diffusion is a critical limiting factor for target capture performance, leading to similar capture rates regardless of the theoretical binding affinities of the capture probes [59]. It is worth making the logical chain underlying this limitation explicit, since it links several parameters discussed separately elsewhere in this review. Pore diameter and porosity together set the tortuosity of the transport path that an analyte must follow to reach a capture probe deep within the film: a narrower or more tortuous pore network lengthens the effective diffusion path relative to the film thickness, which lowers the effective diffusion coefficient of the analyte within the pore relative to its free-solution value. For pores whose diameter approaches the hydrodynamic size of the analyte—for example, for pores of only a few tens of nanometres and a protein or aptamer–target complex of comparable dimension—this restriction is compounded by steric and electrostatic hindrance at the pore mouth, which further reduces analyte accessibility to binding sites located deeper in the film. Because the observed binding kinetics reflect the slower of the two possible rate-limiting steps, once diffusion into the pore becomes slower than the intrinsic association rate of the probe–target pair, the sensor response time and the apparent (as opposed to intrinsic) binding affinity become governed by mass transport rather than by molecular recognition chemistry; this is precisely the situation modelled quantitatively by Arshavsky-Graham et al., who showed that hindered diffusion drives different capture probes toward similar observed capture rates regardless of their intrinsic binding affinities. The net effect on analytical performance is that response time lengthens, and the LOD worsens because a longer time is needed for enough analyte to diffuse to and bind the available probes for the signal to rise above the noise floor.

Further research from Arshavsky-Graham et al. demonstrated an integration of a pSi-based optical aptasensor within a 3D-printed polyacrylate microfluidic platform [60], and significant improvement in protein detection sensitivity. The authors employed a straightforward bonding method using a UV-curable adhesive, which preserves the integrity of the porous structure while enabling effective integration. The 3D-printed microfluidic aptasensor demonstrates an LOD of 0.04 μM, representing a 70-fold improvement compared to the specific non-microfluidic setup tested. Another example involves the application of 3D-printed microfluidic systems featuring passive and active mixing architectures for integration of a pSi sensing platform and detection of biomarker lactoferrin (LF) at clinically relevant concentrations [11]. The authors demonstrate that optimising the pSi nanostructure and its integration can enhance biosensor performance, achieving an LOD of 3 nM.

**Figure 4:**
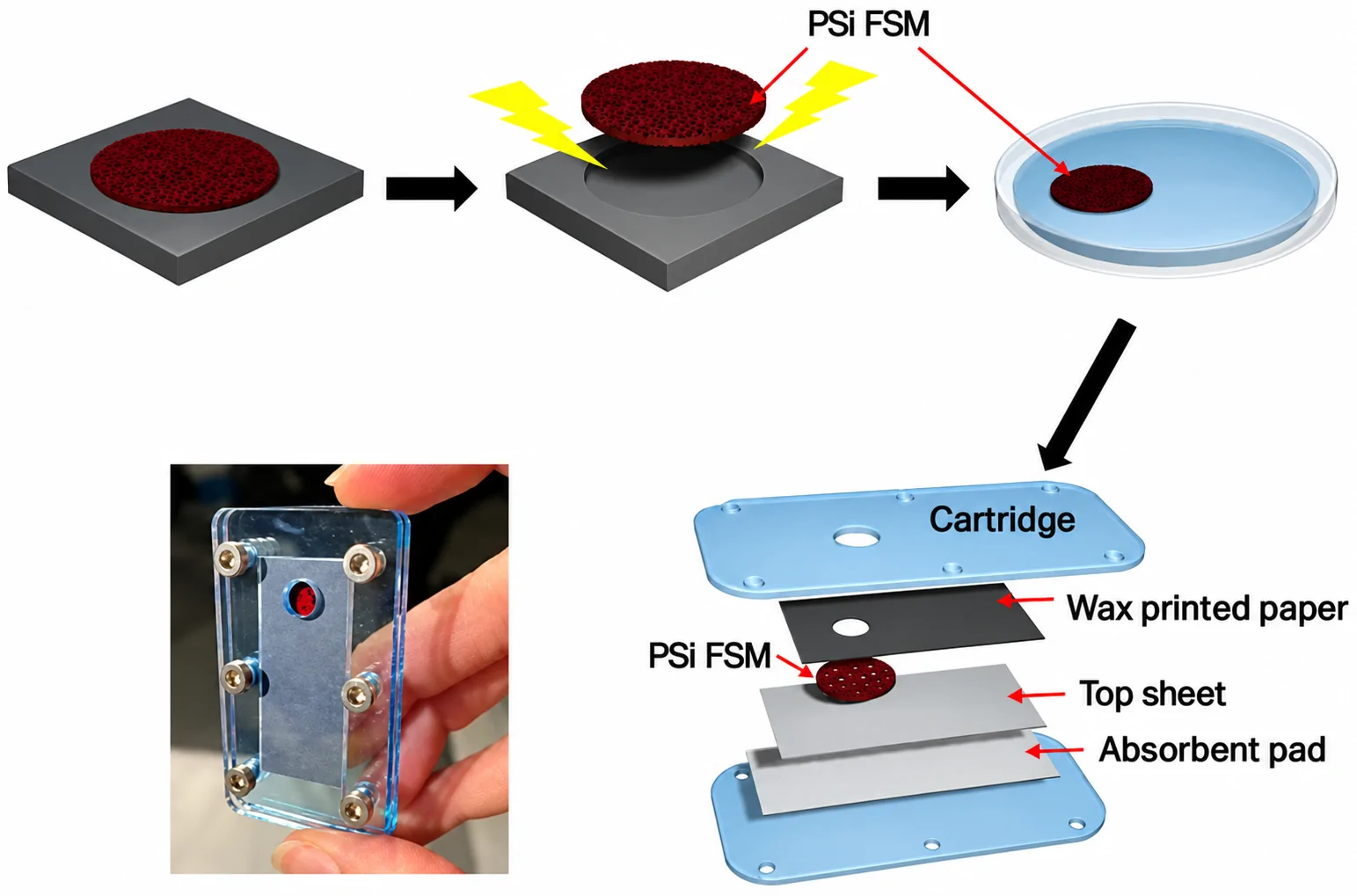
Schematic of the pSi-on-paper fabrication process. Adjusted from Figure 3/Image am4c18940_0003.tif from American Chemical Society Publications, used under CC BY 4.0 [61].

Further advancements in the integration of pSi interferometric transducers are being developed, containing flow-through membranes within lab-on-a-chip (LOC) technology [74]. Huijin An et al. presented a novel flow-through pSi-on-paper sensor platform that significantly enhances molecular transport efficiency, enabling rapid and quantitative detection of streptavidin [61]. The authors developed the flow-through sensor by using open-ended pSi membranes on paper substrates, eliminating the need for external pumps or microfluidic systems. This configuration is an example of free-standing pSi (Figure 4), i.e., the porous layer is fully detached from its parent substrate, yielding a self-supporting membrane with two open faces through which solution can flow directly rather than merely diffusing in from a single side. This resulted in a nearly fourfold increase in detection sensitivity and a response time of approximately 30 min compared to the conventional pSi sensors tested.

The detection of human growth hormone (hGH) is critical due to its significant role in growth and metabolism. Ghiasi Tarzi and colleagues demonstrate a novel pSi-based biosensor utilising RIFTS for real-time hGH detection [62]. The authors fabricated a fluidic optical nanobiosensor by integrating pSi with a CO_2_ laser-machined flow cell, allowing for efficient analyte management and real-time monitoring. Their results indicate a linear response to hGH concentrations ranging from 500 pg/mL to 50 µg/mL, with detection limits of 0.38 ng/mL in buffer and 0.30 ng/mL in human serum, showcasing the sensor’s high specificity and sensitivity.

A high-throughput, fully automated biosensing platform was also demonstrated for efficient COVID-19 detection. The detection method utilises LSPR combined with a pSi microcavity, while robotic arms run the detection process including sample loading, incubation, sensor surface rinsing, and optical measurements using a portable spectrometer [63]. The authors developed a fully automated system that can process up to 384 samples in approximately 30 min, achieving a detection limit of 100 TCID_50/mL_. This method demonstrates sensitivity and a reduced time to results in the tested setup. The biosensor’s design allows for direct detection of viral antigens without complex sample preparation, making it suitable for rapid onsite testing.

**Figure 5:**
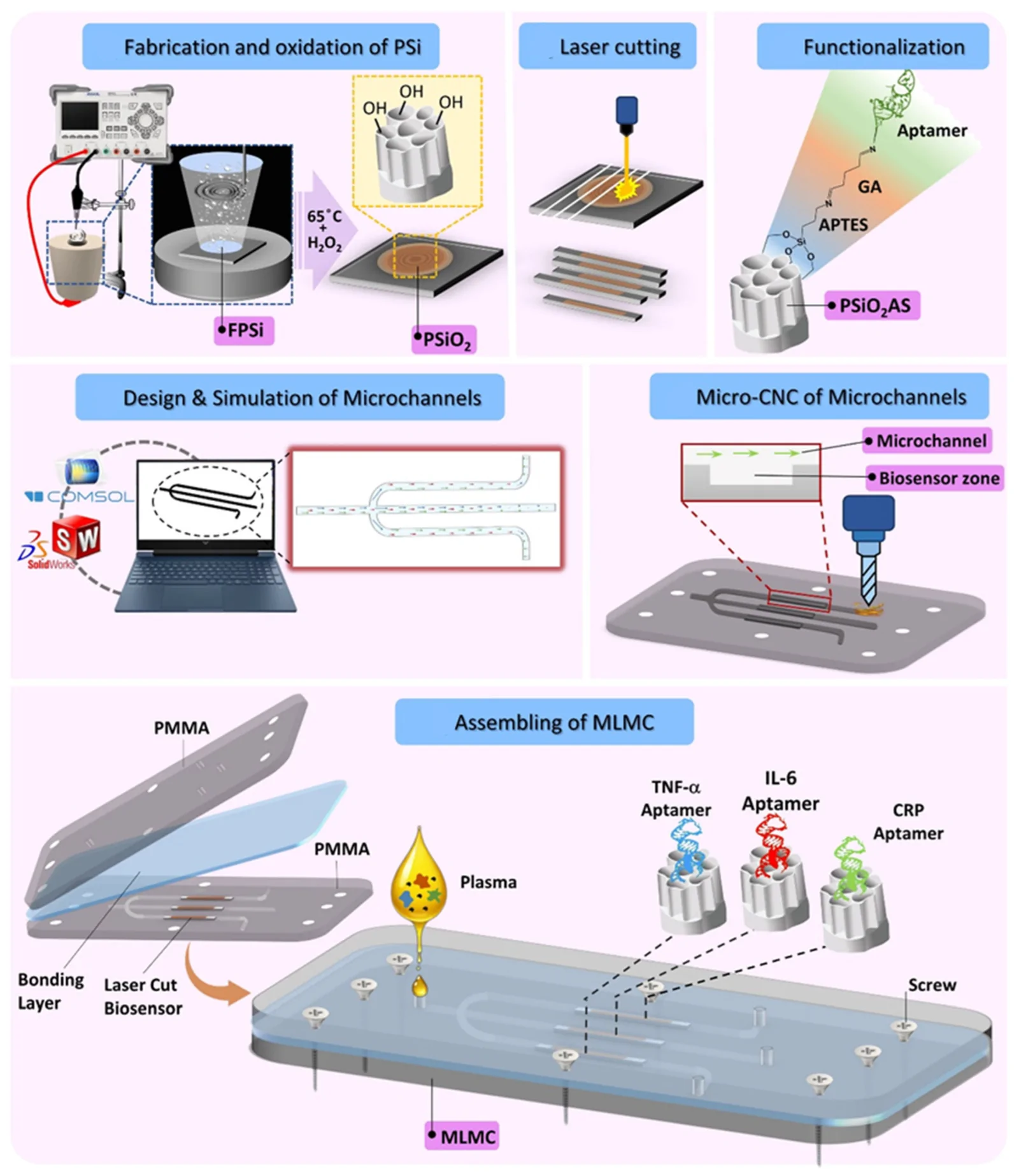
Illustration of the steps from multiplexed microfluidic aptasensor fabrication to multiple analytes detection. Reproduced from Scheme 1/Image 1-s2.0-S2666053925001006-sc1_lrg.jpg from Elsevier, used under CC BY 4.0 [65].

Multiplex biosensing is increasingly important for accurate, rapid diagnosis of complex diseases such as sepsis, which involves multiple circulating biomarkers. Mohammadi et al. report on microfluidic integration of an interferometric porous-silicon aptasensor capable of simultaneous, selective, and label-free detection of key sepsis markers TNF-α, IL-6 and CRP (Figure 5) [65]. The team integrated laser-cut porous-silicon Fabry–Pérot films functionalised with covalently bound aptamers into a reversibly bonded PMMA/PDMS microfluidic chip and used a motorised stage for automated, real-time RIFTS readout.

A label-free NIR-II porous-silicon microcavity (pSiMC) aptasensor that detects insulin in vitro, in plasma, and after subcutaneous implantation in a euthanised mouse was also recently reported [67]. Major findings include reproducible insulin sensing (LOD ≈209 nM for a 7-layer MC), selectivity against common interferents, continuous operation up to 6 h, detection in spiked mouse plasma, and negligible cytotoxicity. The study establishes a foundational platform based on pSiMC and an effective shift-in-wavelength method for implantable, real-time biomarker monitoring.

Taken together, the studies summarised in Section 2 and Section 3 and Table 1 show that optical pSi biosensors have matured to the point of reliably reaching nanomolar-to-picomolar, and in several cases sub-picomolar, LODs for proteins, nucleic acids and whole bacterial cells. This is achieved largely through three complementary strategies: advanced signal processing (e.g., MWPA), engineered photonic architectures (DBRs, microcavities, rugate filters) that sharpen the optical readout, and integration with microfluidics to overcome diffusion limitations. The clearest remaining gaps are the comparatively limited number of studies that validate performance directly in undiluted clinical or environmental matrices rather than buffer, and the continued reliance, in most examples, on a single analyte per chip rather than the multiplexed formats needed for panel-based diagnostics.

## 4. Application of pSi for Electrochemical Sensing

Before turning to specific examples, it is useful to compare the electrochemical and optical pSi platforms directly, since the two rely on fundamentally different sensing mechanisms and therefore offer different trade-offs. Optical pSi biosensors transduce binding events as a change in the effective refractive index of the porous layer, detected as a shift in a reflectance/interference spectrum (RIFTS, DBR/microcavity) or as a change in photoluminescence or Raman intensity; the sensing platform is simply the porous film itself, illuminated externally, and no electrical contact or supporting electrolyte is required at the point of measurement. Electrochemical pSi biosensors, by contrast, require the pSi layer to also function as (or be integrated with) a working electrode: pore-wall conductivity, an ohmic or Schottky back contact to the underlying crystalline silicon substrate, and a defined counter/reference electrode configuration in a supporting electrolyte for the signal to be read. This has direct consequences for performance and instrumentation. Optical readout is inherently label-free, quantitative, and largely insensitive to sample conductivity, and, for well-optimised interferometric and microcavity designs, the examples compiled in Table 1 show it can reach sub-picomolar LOD; however, it generally requires a stable, moderately expensive spectrometer/light-source setup and is more susceptible to non-specific optical scattering from complex biological matrices. Electrochemical readout instead requires only comparatively low-cost, miniaturisable potentiostat electronics, making it attractive for portable and point-of-care formats, and it directly reports on charge-transfer kinetics at the pore walls. The achievable LODs are in the nanomolar-to-picomolar range for most reported protein/DNA electrochemical pSi sensors, as summarised in Table 2, broadly one to three orders of magnitude less sensitive than the best optical microcavity or RIFTS-MWPA aptasensors. Relative to non-pSi biosensor platforms more generally, pSi’s principal advantage in both modalities is its very high internal surface area, which concentrates capture probes and analyte within a compact footprint and can shorten diffusion paths compared with planar SPR or screen-printed electrode sensors. Its principal disadvantage, discussed further in Section 3.4 and in the Future Perspectives section, is that this same porous architecture introduces hindered diffusion and mass-transfer limitations that planar sensors do not suffer from, so that pSi sensors do not automatically outperform well-engineered planar or nanoparticle-based electrochemical and optical biosensors on response time, and the comparison must be made case by case rather than assumed a priori.

### 4.1. Electronic Characteristics of pSi

The pSi derives its electronic behaviour from a band structure that is strongly modified relative to bulk crystalline silicon by quantum confinement, surface depletion, and the large density of surface states created during electrochemical etching [75]. As the pore walls become thinner and the silicon skeleton shrinks to nanometric dimensions, the effective bandgap widens, and the density of states becomes less bulk-like, which helps explain the transition from bulk semiconducting behaviour toward size-dependent optical emission and altered transport. In this regime, carrier motion is no longer governed only by the intrinsic crystal band structure but also by tunnelling barriers between nanocrystallites, oxide layers, and surface trap states.

The electrical properties of pSi are therefore highly sensitive to morphology. In the DC regime, conductivity typically decreases with increasing porosity because the percolation pathways for charge transport become narrower and more interrupted [76]. Carrier transport may proceed by hopping, tunnelling, or thermally assisted hopping between localised states, especially in highly porous or oxidised layers [75]. This means that DC conductivity is often controlled not just by the silicon skeleton itself, but by the connectivity of the network, the degree of surface passivation, and the presence of native oxide or adsorbed species. In moderately porous material, the layer may still conduct reasonably well, but in highly porous films the resistance can rise sharply as the porous network approaches an insulating limit.

In the AC regime, the conductivity and dielectric response are usually more informative than simple DC transport because alternating fields can probe localised carrier motion, interfacial polarisation, and Maxwell–Wagner-type effects [77]. AC conductivity in pSi often shows dispersion with frequency, reflecting a crossover from long-range transport at low frequencies to localised hopping and polarisation loss at higher frequencies. The porous network, together with oxide-filled pore surfaces, can produce strong frequency-dependent dielectric relaxation, which is especially useful for understanding trap states and surface chemistry. This makes AC measurements valuable when DC transport is too dominated by percolation or contact resistance to reveal the underlying physics.

Nonlinear electrical transport is also common in pSi, particularly under strong electric fields or optical excitation [78]. The combination of nanocrystal confinement, surface states, and high internal field gradients can lead to nonlinear current–voltage characteristics, field-assisted hopping, and space-charge-limited conduction. Under illumination, photoconductive gain, carrier trapping, and recombination dynamics can further enhance nonlinearity. In optoelectronic structures, pSi can also show nonlinear optical and electro-optic responses because the porous architecture amplifies local fields and increases interaction with charge carriers and defects [79]. These effects can be exploited for sensing, switching, and integrated photonic devices.

### 4.2. Electrochemical Biosensors Based on pSi

An overview of recent electrochemical biosensors based on pSi and characteristics of the sensing platforms is provided in Table 2.

**Table 2:** Review of electrochemical biosensors based on pSi.

Targets	Method	Wafer Type	Pore Size (PS) and Surface Modification	Limit of Detection	Ref.
hydroquinone (HQ), catechol (CC), resorcinol (RC)	Differential pulse voltammetry (DPV)	p-type	PS 72 ± 15 nm, carbon-stabilised (thermally hydrocarbonised pSi, THCpSi) and thermally carbonised pSi (TCpSi).	1.4 (HQ), 2.4 (CC), 3.1 (RC) μmol/L	[80]
Bacterial RNA fragment (16S rRNA)	DPV	p-type	PS 72 ± 15 nm, THCpSi, ssDNA probes complementary to 16S rRNA	2.3 pM	[81]
bacterial biofilm (*S. epidermidis*)	CV and EIS	p-type	(THCpSi, PS 42 ± 13 nm), (ii) (TCpSi, PS 34 ± 7 nm), (iii) polymer carbonised pSi (PC-pSi, PS: 21 ± 5 nm).	N/A	[82]
insulin, microRNA	DPV and EIS	p-type	PS 22.7 ± 2.5 nm. THCpSi, undecanoic acid, amine-modified bioreceptors (Insulin aptamer or specific DNA capture probes)	Insulin 8.7 pM; microRNA 60–70 pM	[83]
DNA	Electrochemical impedance spectroscopy (EIS)	p-type	PS 60 nm. ssDNA capture probes	1.4 pM	[84]
Calcium	I–V and C–V	p-type	Calmodulin (CaM)	-	[85]
2,4,6-trinitrotoluene (TNT)	Cyclic voltammetry.	p-type	PS N/A, L-Cysteine	0.2 nM	[86]
*E. coli*	Potentiometric	n-type	4–6 μm	0.187 CFU/mL	[87]
Glucose	Conductometric I–V measurement	p-type	PS 1.030 to 1.352 µm. glucose oxidase	0.07 mM (1.26 mg/dL).	[88]
Glucose	Electrical characterisation: I-V curves	p-type	PS 18 ± 3–30 ± 8 nm. Ag nanoparticles	0.59 mM	[89]

To increase the conductivity of pSi, carbon-based materials have emerged as pivotal components on pSi due to their superior electrical properties and surface chemistry. These hybrid materials are synthesised by thermal carbonisation methodology first developed by Salonen et al. [90] The use of carbon-stabilised pSi as a voltammetric sensor was demonstrated for the simultaneous detection of dihydroxybenzene isomers, specifically hydroquinone, catechol, and resorcinol [80]. The authors demonstrated that carbon-stabilised pSi outperforms traditional glassy carbon electrodes in terms of electrochemical performance and sensitivity. Differential pulse voltammetry was employed to assess the electrochemical behaviour of thermally hydrocarbonised (THCpSi) and thermally carbonised (TCpSi) pSi, revealing that TCpSi exhibited superior electron transfer kinetics and reproducibility. Notably, TCpSi enabled the distinct detection of all three isomers, achieving low limits of detection and high recovery rates in spiked tap water samples.

O’Connor et al. report that carbonised pSi can act simultaneously as a scaffold and electrochemical transducer for sensing and characterising bacterial biofilm growth [82]. The authors fabricated three C-pSi variants (hydrophilic TC-pSi, mildly hydrophilic polymer carbonised (PC-pSi), hydrophobic THC-pSi), grew *Staphylococcus epidermidis* biofilms on them, and combined environmental scanning electron microscopy and confocal laser scanning microscopy (ESEM/CLSM) imaging with cyclic voltammetry and electrochemical impedance spectroscopy. This dual-function approach permits real-time electrochemical readout of biofilm coverage, morphology and mass-transfer regime. The results indicate that wettability controls early attachment and architecture of biofilms (TC-pSi yields homogeneous, hospitable biofilms; PC/THC-pSi produce patchy growth), and that CV/EIS signatures reliably distinguish growth stages via linear versus radial diffusion behaviours. The work implies that portable, affordable electrochemical C-pSi sensors could enable point-of-need biofilm surveillance and diagnostics.

**Figure 6:**
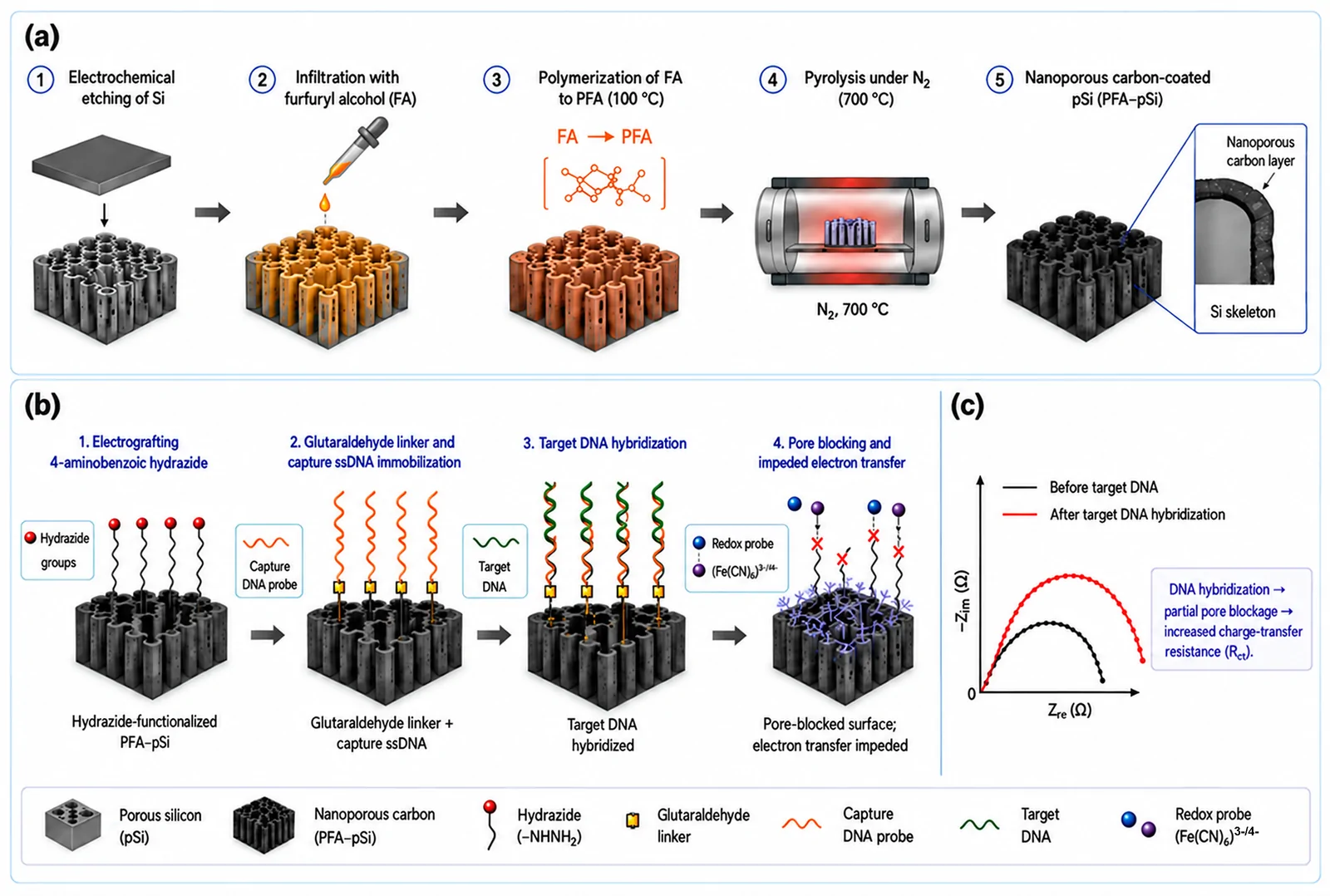
Schematic representation of (**a**) the fabrication of the nanoporous carbon-coated porous silicon (PFA-pSi) electrochemical transducer, (**b**) the sensing concept of its application in label-free DNA sensing and (**c**) electrochemical readout. Target DNA hybridisation induces partial pore blockage, hindering redox-probe transport and increasing the charge-transfer resistance measured by electrochemical impedance spectroscopy. The scheme is based on the sensing strategy reported by Rajendran et al. [84].

Carbonised pSi-based electrochemical platforms were also demonstrated for functionalisation with aptamers and detection of insulin and two selected miRNAs (miR148a and miRlet7g), relevant biomarkers for predicting low milk supply during lactation (Figure 6) [83,84]. On the other hand. Rajendran et al. presented an electrochemical transducer, PFA-pSi, developed through spin coating furfuryl alcohol on preformed pSi, which is further polymerised at 100 °C and thermally carbonised at 700 °C to form a layer of conductive carbon aligned within the pores of pSi, as evidenced by decreasing the pore size from 60 nm to 24 nm [82]. Characterisation of PFA-pSi revealed its highly stable, repeatable, and reproducible electrochemical performance, while also delivering high peak currents due to its porous architecture. The surface is then functionalised with capture DNA probes and applied for electrochemical detection of DNA, exhibiting an LOD of 1.4 pM, significantly outperforming existing sensors based on similar mechanisms. The sensing mechanism relies on the blockage of the pores within the pSi caused upon DNA-base pairing interaction between the surface-functionalised ssDNA probe with target ssDNA. The partial pore blockage of the pores hinders the diffusion of the negatively charged redox species ([Fe(CN)_6_]^3−/4−^), caused mainly by electrostatic repulsions and steric hindrance upon target hybridisation to the ssDNA-modified PFA-pSi.

Another study from the same research group presented carbon-stabilised pSi, obtained through thermal hydrocarbonisation, for assembling a biosensor that enables ultrasensitive, label-free electrochemical detection of bacterial 16S rRNA gene fragments, achieving a limit of detection of 2.3 pM in serum samples [81]. The authors again employed a pore-blockage sensing mechanism and measured signal changes with differential pulse voltammetry (DPV) upon binding the analyte to ssDNA-functionalised THC-pSi.

On the other hand, Sarkar et al. presented a novel electrochemical glucose sensor utilising glucose oxidase physisorbed on macro pSi, demonstrating enhanced sensitivity and responsiveness [88]. The findings reveal that the sensor exhibits a significant increase in current with glucose concentrations up to 1 mM, followed by a decrease at higher concentrations, indicating a complex interaction between glucose adsorption and conductivity. A sensor based on pSi capable of detecting glucose even without functionalisation in the range of 50 mg/dL to 500 mg/dL was also developed [89]. The authors employed a metal-assisted chemical etching (MACE) method to fabricate the pSi layers, followed by electrical characterisation through I-V curves to assess glucose sensitivity. Their findings indicate that the sensor’s performance is optimal with the thinnest pSi layer, demonstrating a clear correlation between glucose concentration and electrical response.

In summary, the electrochemical pSi biosensors reviewed in Section 4 and Table 2 rely predominantly on two transduction principles: pore blockage of a redox probe’s diffusion pathway, detected by EIS or DPV, and direct modulation of the pSi/electrolyte junction’s electrical properties. The methodologies achieve picomolar-to-nanomolar LODs for nucleic acids and bacterial biomarkers using comparatively simple, low-cost instrumentation. As discussed above, their sensitivity is generally more constrained by charge transfer and capacitive background from the semiconducting pSi matrix than their optical counterparts, which is the principal trade-off for their instrumentation and cost advantages. Carbon-stabilised and oxidised pSi surfaces currently represent the most developed route to mitigating this constraint while also improving chemical stability.

## 5. Comparison with Alternative Biosensor Platforms

To assess whether pSi provides a genuine advantage over established biosensor platforms, Table 3 benchmarks the pSi optical and electrochemical sensors reviewed above against four widely used alternatives: planar surface plasmon resonance (SPR) gold-chip biosensors, quartz crystal microbalance (QCM), screen-printed/planar electrochemical electrodes, and ELISA/lateral-flow immunoassays. In terms of sensitivity/LOD, the best-performing pSi optical platforms reach picomolar to sub-picomolar LODs, demonstrating sensitivity comparable to advanced SPR, QCM and electrochemical biosensors. For example, the approximately 0.13 pM LOD reported for miRNA-155 using pSi reflectance sensing is valid, but the exceptional performance depends importantly on Morlet-wavelet phase analysis and spectral-noise suppression [35]. High internal surface area alone does not guarantee superior sensitivity: bulk and hindered intrapore diffusion can prevent efficient access to that area, and pSi capture performance without accelerated transport or signal amplification may be comparable to, or poorer than, planar sensors [59]. Electrochemical pSi sensors are broadly competitive with screen-printed electrodes, with reported advantages usually arising from a particular pore-blockage, surface-modification or amplification architecture rather than from a generic pSi effect.

Response time and analyte transport remain one of the clearest limitations of conventional on-substrate pSi affinity sensors. In addition to transport through the bulk solution, targets must diffuse through tortuous pores, which can produce response times from tens of minutes to approximately 1–2 h [59,61]. Optimised pore geometry, convective microfluidic mixing and open-ended flow-through membranes substantially improve capture and shorten analysis, e.g., to approximately 30 min with a pSi-on-paper flow-through platform [11,61]. Planar SPR, QCM and electrochemical sensors do not experience this additional intrapore-hindered diffusion and tortuosity, although they can still be limited by boundary-layer mass transfer under fast-binding or low-concentration conditions [91].

Stability and reproducibility are surface chemistry- and manufacturing-dependent. As-anodised, hydrogen-terminated pSi has limited oxidative and aqueous stability, whereas thermal oxidation, hydrosilylation and carbon-based stabilisation can markedly improve it [3]. Many reported pSi biosensors are single-use, but single-use is not an intrinsic requirement; regeneration and reuse are simply less established than they are for mature SPR platforms. Gold SPR chips and QCM crystals provide stable transducers, though practical regeneration depends on the immobilised ligand, receptor layer and assay conditions rather than on gold or quartz alone. Likewise, screen-printed electrodes can be highly consistent when industrially manufactured, but ink composition, substrate, printed-layer thickness, curing and subsequent biofunctionalisation can introduce batch and assay variability [92].

pSi interferometric systems can be implemented with relatively simple reflectance optics and compact spectrometers, potentially reducing instrument cost and footprint compared with conventional prism-coupled SPR, while electrochemical pSi was shown to be compatible with compact potentiostats [3]. Nevertheless, portable, fibre-optic and nanoplasmonic SPR formats and compact QCM instruments are also available [91]. Conventional visually interpreted lateral-flow assays retain an advantage in simplicity, speed and cost per test, but generally offer lower quantitative precision and sensitivity. Reader-assisted, amplified and multiplex lateral-flow assays can overcome many of these limitations at the expense of additional reagents or instrumentation [93]. Conventional ELISA remains highly standardised and sensitive but generally requires multiple incubation and washing steps over several hours.

Taken together, pSi is particularly attractive when its tuneable nanostructure, large functionalizable internal surface, optical interference properties and compatibility with compact readout can be exploited while overcoming intrapore mass-transfer limitations. The exceptional LODs generally reflect the combined effects of nanostructure design, surface chemistry, bioreceptor affinity, signal-processing or amplification strategies and efficient analyte transport. pSi may therefore underperform mature planar platforms when rapid transport, repeated regeneration, long-term operational stability or highly standardised manufacturing is the primary requirement.

**Table 3:** Comparison of pSi biosensors with alternative biosensor platforms. Performance ranges are assay-dependent and should not be interpreted as intrinsic platform limits.

Platform	Sensitivity/LOD	Response Time	Analyte Transport	Stability/Reuse	Reproducibility	Instrumentation and Miniaturisation
pSi optical (RIFTS, photonic, QD/FRET)	Reported range spans nM to sub-pM. Exceptional LODs often depend on signal processing, amplification, receptor affinity and assay architecture, not porosity alone [3,35,59]	Typically tens of minutes to ~1–2 h for conventional on-substrate affinity sensors; ~30 min demonstrated with an open-ended flow-through membrane [11,61]	Bulk and intrapore-hindered diffusion/tortuosity can limit capture; improved by optimised pores, convective microfluidics or flow-through membranes [11,59,61].	Surface chemistry-dependent. As-anodised pSi has limited aqueous/oxidative stability; oxidation, hydrosilylation and carbon stabilisation improve it. Many implementations remain single-use.	Moderate; sensitive to wafer properties, etching conditions, porosity/thickness and functionalisation. Process standardisation is important.	Compact reflectance optics and spectrometer; smartphone-compatible formats reported. Potentially lower cost/footprint than conventional prism SPR [61]
pSi electrochemical	Often pM to nM for reported nucleic-acid and biomarker assays; performance depends strongly on electrode chemistry, receptor, pore-blockage design and amplification.	Usually minutes; pore infiltration and pore-blockage kinetics may govern equilibration.	Intrapore diffusion can limit transport, as in optical pSi; convection and larger/open pores can improve access.	Surface chemistry-dependent; carbon stabilisation and oxidation improve robustness. Many demonstrations are single-use, but reuse is not intrinsically excluded.	Moderate; wafer/etching variability and surface modification affect baseline and response.	Compatible with low-cost portable potentiostats; highly miniaturizable.
Planar SPR (gold chip)	Commonly pM-nM for biomolecular assays; optimised or amplified formats may reach sub-pM concentrations. Strongly assay-dependent [91,94].	Real-time response commonly seconds to minutes, although association/incubation time remains assay-dependent.	No intrapore tortuosity, but boundary-layer mass transfer can still limit fast-binding or low-concentration assays [91].	Stable gold transducer; regeneration/reuse often possible, but governed by ligand, receptor and assay chemistry rather than gold alone.	High at instrument/chip level; biofunctionalisation introduces assay-dependent variability.	Conventional prism optics are comparatively complex/costly; portable, fibre-optic and nanoplasmonic formats also exist [91].
Quartz crystal microbalance (QCM)	nM to pM, and lower with amplification, depending on target, coating and assay format [94].	Real-time mass readout; practical binding/incubation times range from minutes upward and are assay-dependent.	No pSi-like intrapore limitation on a planar crystal, although boundary-layer transport can still be limiting.	Quartz transducer can be cleaned/reused; functional coating and bioreceptor stability determine assay regeneration.	High at transducer level; coating and biological layers introduce assay-to-assay variability.	Requires oscillator or impedance electronics; compact and portable implementations are feasible.
Screen-printed/planar electrochemical electrode	nM to pM or below depending on electrode modification, receptor and amplification [92].	Usually minutes; planar designs avoid pSi intrapore hindrance, although solution-phase transport and binding kinetics can still limit response.	No pSi pore tortuosity unless an intentionally porous/nanostructured modification is added.	Commonly disposable; stability is governed by the printed material, biofunctional layer, fouling and storage conditions [92].	Moderate-to-high for industrial production; variability can arise from inks, substrate, printing/curing and post-print modification [92].	Low-cost portable potentiostat; highly miniaturizable; single-use strips are common.
ELISA/lateral-flow immunoassay	ELISA commonly pM-nM; lateral-flow sensitivity is broad and format-dependent. Advanced labels/amplification can markedly improve LFA sensitivity [93].	ELISA: usually hours and multi-step. Lateral flow: usually minutes.	Bulk-solution transport (ELISA) or capillary flow (LFA); no pSi-like intrapore tortuosity.	Reagent- and storage-dependent; single-use by design.	High for standardised ELISA kits; moderate-to-high for LFA, with lot, matrix and readout dependence.	ELISA usually requires a plate reader/lab workflow. Visual LFA needs minimal instrumentation; quantitative/multiplex LFA generally requires a reader [93].

## 6. Future Perspectives

pSi continues to demonstrate exceptional potential as a multifunctional material for next-generation biosensing technologies. Its tuneable nanostructure, compatibility with silicon microfabrication, large surface area, and versatile optical and electrochemical properties position it as a promising platform for highly sensitive and miniaturised sensing systems. Nevertheless, several scientific and technological challenges must be addressed to fully realise the transition of pSi-based biosensors from laboratory-scale demonstrations to practical commercial and clinical applications.

One of the most important future directions involves improving the long-term chemical and mechanical stability of pSi structures. Although the high surface reactivity of pSi is advantageous for biomolecule immobilisation, it also makes the material susceptible to oxidation, dissolution, and degradation under physiological and environmental conditions. Future research should therefore focus on developing robust surface passivation and functionalisation strategies that preserve sensor performance while enhancing stability and reproducibility. Surface coatings based on polymers, metal oxides, graphene derivatives, and hybrid organic–inorganic layers are expected to play a key role in improving durability and resistance to biofouling. Namely, freshly etched pSi is intrinsically fragile: the Si-H-terminated surface is metastable and undergoes gradual oxidative dissolution when immersed in aqueous or physiological media, so a pSi photonic crystal used for label-free optical sensing typically cannot be regenerated and reused for repeated measurement cycles the way a planar SPR chip can, and progressive pore widening and mechanical weakening over successive exposures is a recognised failure mode rather than an occasional artefact. A second, related limitation is that once a pSi surface has been functionalised with a bioreceptor layer (silanes, antibodies, aptamers, or DNA probes), that specific layer generally cannot be stripped and the surface “cleaned” and re-functionalised without damaging the underlying porous nanostructure, so most pSi biosensors reported to date are single-use, disposable devices rather than reusable ones. Several strategies have nonetheless begun to overcome these constraints. Thermal or chemical stabilisation of the pSi surface, for example, thermal carbonisation/hydrocarbonisation to form Si-C-terminated, hydrolytically stable pSi, or controlled thermal oxidation to SiO_2_, markedly slows dissolution. This methodology has already enabled the carbon-stabilised, ultrasensitive electrochemical rRNA biosensor to retain stable baseline signals over extended incubation and measurement periods, illustrating that surface stabilisation is a realistic route to improved pSi biosensor durability.

Another critical challenge concerns the reproducibility and scalability of electrochemical etching processes. Small variations in wafer properties, electrolyte composition, current density, and cell configuration can significantly alter pore morphology and sensing characteristics. Standardisation of fabrication protocols and deeper understanding of pore formation mechanisms are essential for achieving reliable large-scale production. Integration of advanced process monitoring, automation, and machine learning-assisted optimisation may enable precise control over pore architecture and improve batch-to-batch consistency in future manufacturing processes.

To make the directions above more concrete, we link each open problem to the proposed addressing strategy, and to our assessment of its current maturity (established, emerging or conceptual). Chemical/mechanical instability is addressed by surface passivation (thermal carbonisation/oxidation, polymer or metal-oxide coatings), which we regard as established for improving hydrolytic stability, though multi-cycle reusability of a single sensor remains emerging. Batch-to-batch fabrication variability is addressed by etching-protocol standardisation and in-line process monitoring, established for basic etching parameters but emerging for automated, closed-loop control. Mass-transfer limitation is addressed by microfluidic/lab-on-a-chip integration and macroporous or thin-film architectures, both established, given the multiple worked examples with quantified LOD improvements discussed in Section 3.4. Multiplexed, simultaneous biomarker detection is addressed by patterned or laser-cut multi-region pSi chips, established for two-to-three analyte panels but emerging for larger panels. AI-assisted signal interpretation is—from the evidence reviewed here—still largely conceptual for pSi biosensors specifically, since only isolated examples (e.g., machine learning-assisted RIFTS classification) were identified, even though the underlying methods are mature in other analytical contexts. Implantable/theranostic use is conceptual and emerging, supported so far mainly by biocompatibility and degradation studies rather than by validated in vivo biosensing performance.

Combining pSi with nanomaterials such as metallic nanoparticles, quantum dots, carbon nanotubes, graphene, and conductive polymers has already been shown to lower the LOD by one to several orders of magnitude relative to unmodified pSi, as in the SERS-active Ag/Au-pSi platforms and the QD-labelled Bragg mirror and microcavity sensors that combine refractive index and photoluminescence readout. Incorporation of plasmonic and photonic nanostructures within pSi matrices, following the SPR-nanohole-array and SERS examples discussed above, extends this same signal amplification approach to additional label-free optical formats.

The integration of pSi biosensors with microfluidic and lab-on-a-chip technologies is, from the evidence reviewed in Section 3.4, the most effective strategy demonstrated to date for overcoming the mass-transfer limitations intrinsic to porous architectures: documented examples include a 70-fold LOD improvement for a 3D-printed microfluidic aptasensor, a nearly fourfold sensitivity increase with a ~30 min response time for a flow-through pSi-on-paper platform, and simultaneous multiplexed detection of TNF-α, IL-6 and CRP on a single PMMA/PDMS-integrated chip. Future point-of-care devices are expected to build directly on these validated configurations, combining the automated sample preparation and multiplexed detection formats already demonstrated at the single-chip level with the wireless readout and portable spectrometer or potentiostat electronics used in the automated COVID-19 and RIFTS platforms discussed above.

Machine learning has so far been applied to pSi biosensor data in only a small number of reported cases, most notably for classifying and quantifying proteins from reflectance spectra without dedicated capture agents, achieving 100% accuracy for known concentrations and 87.5% accuracy for unseen concentrations. Extending such approaches to noise reduction and analyte classification for the RIFTS, EIS and DPV datasets generated by the sensors is a specific, testable next step, building on the demonstrated gains from Morlet wavelet phase analysis for signal denoising. Coupling this signal-processing capability with wearable or IoT-enabled readout electronics would further extend capabilities for continuous, remote monitoring.

In biomedical applications, extending the multiplexed detection already demonstrated for two-to-three analyte panels, such as the sepsis biomarker panel TNF-α/IL-6/CRP and the multiplex bacterial SERS assay discussed above, to larger biomarker panels could be a viable option for early disease diagnosis and pathogen identification. For implantable and theranostic applications, the NIR-II pSiMC insulin aptasensor validated in plasma and after subcutaneous implantation provides concrete proof of concept. Though further investigation of the biocompatibility and biodegradability of pSi over longer implantation periods is needed before theranostic platforms that combine sensing with drug delivery can be evaluated beyond the laboratory scale.

Despite the remarkable progress achieved thus far, commercialisation of pSi biosensors still requires addressing issues related to device packaging, operational stability, regulatory approval, and cost-effective mass production. Collaboration among materials scientists, chemists, engineers, clinicians, and industrial partners will therefore be essential for translating laboratory-scale innovations into practical technologies.

Overall, the advances in nanofabrication, surface engineering, microfluidics, and computational analysis discussed throughout this review have already delivered measurable improvements in the sensitivity, stability, and integration of pSi biosensors. Translating these individually validated strategies into combined, standardised platforms, rather than developing further isolated proof-of-concept devices, is the main remaining task for establishing pSi as a routinely used material in healthcare, environmental monitoring, and industrial sensing applications.

## Figures and Tables

**Table 1:** Overview of recent research studies based on pSi optical biosensors.

Targets	Detection Method	Wafer Type	Pore size (PS) and Surface Modification	Limit of Detection	Ref.
*Bacillus cereus* lysate	RIFTS	p-type	PS 41 nm	10^5^ CFU/mL	[42]
*Bacillus cereus* lysate	RIFTS	p-type	pSi 55.5 ± 25.4 nm pSi/TiO_2_: 33.5 ± 15.1 nm	10^3^ CFU/mL	[43]
*E. coli* heat-labile enterotoxin subunit (LTB)	RIFTS	p-type	PS 13–70 nm, thermoresponsive glycopolymer with recognition motifs (Lac and GM1os)	0.135 μM	[44]
*E. coli*	reflectometric, FFT peak attenuation	p-type	23.5, 41.7 and 52.9 nm, goat anti-rabbit secondary antibodies (s-ab)	2 CFU/mL	[45]
*E. coli*, *S. aureus* and *B. cereus* Multiplex detection	SERS (surface-enhanced Raman scattering).	Not specified	PS 14 ± 2 nm. Si wafers patterned via SU-8 photolithography and etched. Functionalised with Ag and s-ab (anti-rabbit secondary antibodies).	*E. coli* 5 CFU/mL, *S. aureus* 5 CFU/mL, and *B. cereus* 4 CFU/mL	[31]
Malachite green	SERS	p-type	Silver nano-dendritic structures on pSi	10^−8^ M	[32]
Rhodamine 101, methyl parathion MP)	SERS	p-type	Silver nanoparticles (AgNPs) deposited onto pSi substrates using an immersion plating technique.	Rhodamine 101: 1.0 × 10^−13^ M; MP: 1.0 × 10^−10^ M	[33]
mycotoxins (Ochratoxin A, Fumonisin B1 and Alfatoxin B1)	SERS, portable Raman device	p-type	Silver-coated pSi (Ag-pSi) modified with anti-target mycotoxin aptamer (aptamer/DTNB (Raman tag)/Ag-pSi surface).	ochratoxin A: 0.922 ppb, fumonisin B1: 0.547 ppb and aflatoxin B1: 0.008 ppb	[30]
Glucose	RIFTS	p-type	PS 18 nm–1.7 μm. Physisorbed glucose oxidase (GOX) enzyme	LOD: N/A, Sensitivity: 0.41 mM	[46]
Heat shock protein 70 (HSP70).	RIFTS	p-type	PS 40 ± 20 nm, Anti-HSP70	1290 ± 160 ng/mL	[47]
β-lactoglobulin (β-lg)	Fluorescence and RIFTS	p-type	PS 30 nm. pSi: beta-lg. Quantum dots (QDs) CdSe/ZnS: beta-lg antibodies	0.12 ng/mL	[48]
Target DNA	Fluorescence	p-type	PS 30 nm, DNA probe	88 pM	[49]
CdSe/ZnS QD-labelled probe DNA	angle-spectrum, pSi microcavity	p-type	APTES, glutaraldehyde, probe DNA	13.25 pM	[50]
Protein A, bovine serum albumin (BSA), functionalised biotin and rabbit IgG	RIFTS and SPR	p-type	PS 22 ± 4 nm, plasmonic Au nanohole arrays with diameter of 173 ± 30 nm.	LOD N/A, Sensitivity: 386.3 ± 5.4 nm/RIU	[51]
*Botulinum* neurotoxins (BoNT)	RIFTS	p-type	PS 70 ± 20 nm, BoNT-C toxoid.	4.8 pg/mL.	[52]
*Botulinum* neurotoxin C (BoNT/C)	RIFTS	p-type	PS 70 ± 15, BoNT/C Toxoid or synaptosomal-associated protein (SNAP25B)	4.24 pg/mL.	[53]
His-tagged tyrosinase	RIFTS	p-type	PS 35–65. Anti-his-tag aptamer (6H7), antibodies	5 μM	[54]
Carnosine, adenosine triphosphate (ATP)	RIFTS	p-type	PS 60 nm. Iminodiacetic acid (IDA) as a metal-ion chelator, Cu^2+^/Zn^2+^ for carnosine detection and Fe^3+^ for ATP	Carnosine: 25 µM; ATP 30 µM	[55]
Human cardiac troponin T (cTnT)	RIFTS	n-type	PS 50–250 nm, 10-undecenoic acid, PNA probes	30 pg/mL	[56]
Cancer biomarker AGR2	RIFTS, Morlet Wavelet Phase Analysis (MWPA)	p-type	PS 40–60 nm, anti-AGR2 aptamers.	MWPA: 15 nM, RIFTS: 0.2 μM	[36]
miRNA-155	Reflectance—MWPA	p-type	PS 11 ± 2 nm, amine-modified ssDNA capture probe	MWPA: 0.13 pM, RIFTS: 0.72 pM	[35]
Chikungunya virus E2 protein	RIFTS	p-type	PS 40–60 nm. E2-binding peptide.	1 µM	[57]
Human papillomavirus 16 and 18 (HPV16 and HPV18)—DNA	RIFTS	p-type	PS 20.5 ± 6 nm, ssDNA oligonucleotides of HPV16 and HPV18	25 μM	[58]
Proteins	RIFTS	p-type	PS 50 nm. Aptamer and antibody	1 μM	[59]
His-tagged proteins	RIFTS	p-type	PS 50 nm, aptamer	40 nM	[60]
Lactoferrin (LF)	RIFTS	p-type	PS 50 nm and 80 nm. Anti-LF aptamers	50 nM	[11]
Streptavidin (SA)	RIFTS	p-type	PS 47 nm, 34 nm. Sulfo-NHS-biotin	10 μM	[61]
Human growth hormone (hGH)	RIFTS	p-type	PS 20–25 nm, anti-hGH	0.38 (buffer) and 0.30 ng/mL (serum).	[62]
SARS-CoV-2	RIFTS, Localised Surface plasmon resonance (LSPR)	p-type	Engineered trimeric angiotensin converting enzyme-2 (T-ACE2) protein	100 TCID_50/mL_,	[63]
Rabbit IgG and *Erythrina cristagalli* lectin (ECL)	LSPR	p-type	Protein A, asialofetuin	0.4 μg/mL for rabbit IgG, 0.2 μg/mL for ECL	[64]
Tumour necrosis factor-alpha (TNF-α), interleukin-6 (IL-6), and C-reactive protein (CRP)	RIFTS	p-type	10–105 nm, oxidised pSi, APTES-GA-aptamer modification. The modified pSi was integrated into the microfluidic platform.	4.75 pM (TNF-alfa), 7.36 pM (IL-6), 9.19 nM (CRP)	[65]
IL-6	RIFTS	n-type	PS 50–250 nm, ~5 nm molecularly imprinted polymer (MIP) tailored to selectively bind IL-6.	13 nM	[66]
Insulin	microcavity resonance shift (reflectance minimum)	p-type	Anti-insulin DNA aptamer as the capture probe.	209 nM	[67]
Acetamiprid (AA) and profenofos (PF)	Fluorescence	p-type	Single-stranded DNA (ssDNA) aptamers.	AA 2.64 nM; PF 1.05 nM	[68]
Protein (OVA, Avidin, BSA)	RIFTS, machine learning	p-type	PS 12.0 nm, 15.1 nm, 17.3 nm.	300 nM	[69]
CagA of *H. pylori*.	Reflectometric, Tamm Plasmon Polariton	p-type	PS 20–30 nm. Cag A antibody	0.05 ng/mL	[70]
Vaginal pH	Fluorescence, phone readout	-	PS 6.9 ± 2.7 and 35.9 ± 13.6 nm. Fluorescent polyelectrolytes.	LOD N/A Tested pH: 3–7.5	[71]
Co^2+^, Cu^2+^ and Hg^2+^ ions	Photoluminescence	Not reported	Nucleophilic ligand	3.14 × 10^−9^ M (Co^2+^), 7.43 × 10^−9^ M (Cu^2+^), and 8.21 × 10^−4^ M (Hg^2+^).	[72]
Pb^2+^	RIFTS	p-type	PS 30–50 nm, DNAzyme	0.49 ppb (2.4 nM)	[73]

## Data Availability

No new data were created or analyzed in this study. Data sharing is not applicable to this article.
